# High-Sensitivity Cardiac Troponin T and I Discordance in Patients Receiving Immune Checkpoint Inhibitors

**DOI:** 10.1016/j.jaccao.2026.07.012

**Published:** 2026-10-20

**Authors:** Anis Ismail, Alexi Vasbinder, Bobby Bobby, McKenna de Bever, Aidan Vosooghi, Valeria Pena-Valentin, Lihua Shu, Sujay Kalathoor, Ramya Elangovan, Rachel Jones, Julia Skibniewska, Ian Pizzo, Tonimarie Catalan, Theresa Farhat, Pennelope Kunkle, Monika Leja, Salim S. Hayek

**Affiliations:** a Department of Internal Medicine, University of Texas Medical Branch, Galveston, Texas, USA; b School of Nursing, University of Washington, Seattle, Washington, USA; c John Sealy School of Medicine, University of Texas Medical Branch, Galveston, Texas, USA; d Department of Internal Medicine, Michigan Medicine, University of Michigan, Ann Arbor, Michigan, USA; e Division of Cardiology, Michigan Medicine, University of Michigan, Ann Arbor, Michigan, USA

**Keywords:** biomarkers, biomarker surveillance, cardiac troponin, cardio-oncology, immune checkpoint inhibitors, immunotherapy, myocarditis, myositis

## Abstract

**Background:**

Serial troponin monitoring is used during immune checkpoint inhibitor (ICI) therapy, but guidelines do not specify whether high-sensitivity cardiac troponin I (hs-cTnI) or T (hs-cTnT) should be measured. Unlike cardiac-specific hs-cTnI, hs-cTnT can rise from noncardiac sources.

**Objectives:**

This study sought to determine the prevalence and temporal stability of hs-cTnT/hs-cTnI discordance, identify clinical correlates, and evaluate prognostic implications in ambulatory ICI-treated patients.

**Methods:**

In this prospective single-center cohort, patients starting ICI therapy underwent paired hs-cTnT (Roche Elecsys) and hs-cTnI (Siemens Atellica) measurements in the same banked samples at baseline and 1 month. Patients with adjudicated ICI-associated myocarditis or myositis were excluded by design.

**Results:**

Among 433 patients with paired baseline measurements, hs-cTnT was elevated in 37.9% and hs-cTnI in 1.8%; 158 (36.5%) were discordant (elevated hs-cTnT, normal hs-cTnI), and 267 (61.7%) had normal levels of both (κ = 0.036). Among 329 patients with paired data at baseline and 1 month, discordance prevalence was unchanged (38.3% vs 38.0%; McNemar *P* > 0.99). Independent correlates were older age, male sex, and chronic kidney disease (all *P* < 0.05). Over a median follow-up of 30.1 months (reverse Kaplan-Meier), elevated hs-cTnT was associated with all-cause mortality (adjusted HR: 1.35; 95% CI: 1.00-1.81; *P* = 0.048); only 8 of 231 deaths (3.5%) were cardiac.

**Conclusions:**

In ambulatory patients with cancer receiving ICI therapy, over one-third had elevated hs-cTnT with normal hs-cTnI: a stable, nonspecific pattern present before ICI exposure and consistent with noncardiac sources rather than acute cardiac injury. hs-cTnI is the more cardiac-specific assay; paired baseline measurement of both troponins may help contextualize later elevations when myocarditis is suspected.

Immune checkpoint inhibitors (ICIs) have transformed cancer treatment but can trigger cardiovascular and musculoskeletal immune-related adverse events.^1^^,^^2^ ICI-associated myocarditis, the most feared cardiovascular complication, carries case-fatality rates of 25% to 50%.^3^, ^4^, ^5^ Although overt myocarditis affects approximately 1% of ICI-treated patients, its high case-fatality rate has driven the adoption of serial troponin monitoring during ICI therapy to enable early identification of myocarditis and timely intervention before fulminant presentation.^6^^,^^7^

The 2022 European Society of Cardiology cardio-oncology guidelines recommend baseline troponin measurement in all patients (Class I) and serial troponin measurement during ICI therapy (Class IIa).^8^ By contrast, the American College of Cardiology addresses troponin measurement in the evaluation of suspected myocarditis rather than for routine surveillance,^9^ and the American Society of Clinical Oncology found insufficient evidence to recommend routine baseline or serial troponin testing;^10^ none specify whether high-sensitivity cardiac troponin I (hs-cTnI) or high-sensitivity cardiac troponin T (hs-cTnT) should be used. Assay specificity is central to this diagnosis: a positive result can trigger advanced cardiac imaging, interruption of potentially life-saving cancer therapy, and high-dose corticosteroids, with escalation to targeted immunosuppressants in severe cases.^11^, ^12^, ^13^ The chosen assay must therefore maximize cardiac specificity without sacrificing sensitivity for acute myocardial injury.

Although both assays are gold standard biomarkers for myocardial injury, they differ in tissue specificity.^14^^,^^15^ Cardiac troponin I (*TNNI3*) is expressed exclusively in cardiac myocytes with no documented skeletal re-expression.^16^^,^^17^ Cardiac troponin T (*TNNT2*) can rise because of skeletal muscle re-expression in myopathies and muscle wasting,^18^, ^19^, ^20^, ^21^ reduced renal clearance in chronic kidney disease (CKD),^22^^,^^23^ and chronic myocardial turnover.^24^

ICI therapy creates particular conditions for hs-cTnT/hs-cTnI discordance. ICI-associated myositis^25^ is an uncommon immune-related adverse event, and 25% to 46% of those with ICI myocarditis have concurrent skeletal myopathy driven by clonal T-cell infiltration of both tissues.^5^^,^^6^^,^^26^, ^27^, ^28^, ^29^ In clinically manifest ICI myotoxicity, hs-cTnI normalizes within a median of 17 days, whereas hs-cTnT remains elevated for months, reflecting *TNNT2* re-expression in regenerating skeletal muscle.^7^^,^^30^ Whether ICI therapy also induces hs-cTnT elevation in the asymptomatic majority who undergo serial surveillance is unknown.

We conducted a prospective cohort study with paired hs-cTnI and hs-cTnT measurements at baseline and 1 month in ambulatory patients starting ICI therapy, excluding patients adjudicated to have ICI-associated myocarditis or myositis, to isolate patients undergoing routine surveillance. Our objectives were to determine the prevalence and temporal stability of hs-cTnT/hs-cTnI discordance, identify clinical correlates, and evaluate prognostic implications for all-cause mortality.

## Methods

### Study design and setting

This was a prospective, single-center, observational cohort study nested within the Michigan Immunotherapy Cardiovascular Research Study at the University of Michigan (Rogel Cancer Center). Consecutive ambulatory adults with a pathologically confirmed malignancy starting a new course of ICI therapy were enrolled between March 2023 and December 2024, excluding those with clinically adjudicated ICI-associated myocarditis or myositis. The study was approved by the University of Michigan Institutional Review Board (HUM00231167) with a waiver of informed consent for analysis of residual biospecimens and was conducted in accordance with the Declaration of Helsinki.

### Study group

Eligible patients were adults (≥18 years) with a solid or hematologic malignancy starting ICI therapy (antiprogrammed cell death protein 1 [PD-1], antiprogrammed death-ligand 1 [PD-L1], anticytotoxic T-lymphocyte–associated protein 4, or combination). Patients with adjudicated ICI-associated myocarditis (n = 1) or myositis (n = 1) were excluded by design. Baseline characteristics of excluded vs analyzed patients appear in Supplemental Table 1.

### Data collection

Clinical characteristics, comorbidities, cancer type and stage, ICI regimen, immune-related adverse events, and laboratory values were abstracted by chart review and entered into the Research Electronic Data Capture database. Vital status was ascertained from medical records, the National Death Index, and the Michigan Death Index. Cause of death was adjudicated as cardiac (International Classification of Diseases, Tenth Revision, codes I00-I52) or noncardiac by 2 investigators blinded to troponin status; discrepancies were resolved by consensus. The date last known alive was used for censoring.

### Troponin measurements

Residual blood from clinically indicated blood draws was banked at baseline (pretherapy), approximately 1 month, and approximately 6 months, corresponding to routine clinical visits. Both assays were performed on the same sample by technicians blinded to clinical data: hs-cTnT with the Roche Elecsys fifth-generation assay (Roche Diagnostics) on the cobas platform and hs-cTnI with the Siemens Atellica IM assay (Siemens Healthineers).

### Troponin definitions

Troponin elevation was defined by the manufacturers’ overall 99th-percentile upper reference limits (URLs): hs-cTnT ≥14 ng/L (Roche Elecsys; limit of detection [LOD], 6 ng/L) and hs-cTnI ≥45.2 ng/L (Siemens Atellica; LOD, 2.5 ng/L). Both assays met the <10% coefficient of variation criterion at the 99th percentile. Sex-specific thresholds (hs-cTnT, 22 ng/L for men and 14 ng/L for women; hs-cTnI, 53 ng/L for men and 35 ng/L for women) were applied in a sensitivity analysis. Values below the LOD were replaced with the LOD value.

Among patients with paired measurements, a 4-group concordance classification was assigned at each timepoint: 1) both normal; 2) TnT+/TnI− (elevated hs-cTnT with normal hs-cTnI; hereafter “discordant”); 3) both elevated; and 4) TnI+/TnT− (“reverse discordant”).

### Outcomes

The primary outcome was baseline prevalence of TnT+/TnI− discordance. Secondary outcomes were 1) temporal stability from baseline to 1 month, 2) clinical correlates of baseline discordance, and 3) all-cause mortality from the baseline draw. Six-month biomarker assessments were exploratory (Supplemental Figure 3, Supplemental Table 2). With only 8 cardiac deaths, causes of death are described but were not formally analyzed.

### Derived variables

Cancer types were collapsed from 22 Research Electronic Data Capture categories into 6 analytical groups: melanoma, lung, head and neck/cutaneous squamous cell carcinoma, genitourinary, gastrointestinal/hepatobiliary, and other. ICI agents were classified as anti-PD-1, anti-PD-L1, anticytotoxic T-lymphocyte–associated protein 4, combination, or other.

### Statistical analysis

Baseline characteristics were summarized as median (25th-75th percentiles [Q1-Q3]) for continuous variables and n (%) for categorical variables and were compared using the Kruskal-Wallis or Fisher exact test (Table 1).

Troponin distributions were visualized with violin-and-box plots on a log10 scale. McNemar’s test compared marginal proportions of TnT and TnI elevation; Cohen’s kappa (95% CI from 1,000 bootstrap resamples) quantified interassay agreement. Stability of discordance from baseline to 1 month was assessed by McNemar’s test among patients with paired data; the 6-month timepoint was reserved for exploratory analyses (Supplemental Figure 3, Supplemental Table 2).

A linear mixed-effects model was fit to baseline and 1-month observations in the paired-baseline cohort, with natural-logarithm–transformed troponin (log_e) as the outcome, a random intercept for patient, and fixed effects for timepoint, biomarker, their interaction, and covariates (age, sex, CKD, and cancer category). The timepoint–by–biomarker interaction assessed whether hs-cTnT and hs-cTnI trajectories diverged. Fixed-effect estimates are presented as the regression coefficient (β) with 95% CI and as fold differences in troponin concentration for clinical interpretability.

Multivariable logistic regression modeled TnT+/TnI− vs both normal at baseline (both elevated [n = 6] and reverse discordant [n = 2] were too infrequent to model). The parsimonious model included age (per 10 years), sex, body mass index (per 5 kg/m^2^), CKD, cancer category, and ICI class. Linearity was assessed with restricted cubic splines and collinearity with generalized variance inflation factors. A sensitivity model added coronary artery disease, heart failure, hypertension, diabetes, and smoking status. Model results are presented as ORs with 95% CI and discrimination as the area under the receiver operating characteristic curve (C-statistic) with DeLong 95% CI. Melanoma was the reference category for cancer type in all logistic and Cox models.

Median follow-up was estimated using the reverse Kaplan-Meier method. The Kaplan-Meier curves were stratified by hs-cTnT elevation and concordance group. Prespecified Cox proportional hazards models for hs-cTnT elevation, hs-cTnI elevation, and concordance group were fit in 3 specifications: unadjusted; age- and sex-adjusted; and fully adjusted for age, sex, cancer category, CKD, coronary artery disease, heart failure, and diabetes. An additional model added continuous log-transformed troponins to assess attenuation. Unadjusted and age- and sex-adjusted models are provided in Supplemental Table 5, and the fully adjusted models are provided in Table 2. Cox results are presented as the HR with 95% CI and discrimination as Harrell’s C-statistic (global concordance) with 95% CI. The proportional hazards assumption was evaluated using Schoenfeld residuals. Given only 8 cardiac deaths, cause-specific analyses were exploratory.

**Table 2:** Cox Proportional Hazards Models for All-Cause Mortality

	HR	95% CI	*P* Value	C-Statistic
Model 1: hs-cTnT, fully adjusted				0.608 (0.571-0.645)
hs-cTnT elevated (≥14 ng/L)	1.35	1.00-1.81	0.048	
Age (per y)	1.01	0.99-1.02	0.3	
Women	0.81	0.61-1.08	0.2	
Melanoma (reference)	1.00	Reference	—	
Lung	1.62	0.89-2.94	0.1	
Head and neck/cutaneous SCC	2.21	1.26-3.88	0.006	
Genitourinary	1.82	1.04-3.19	0.035	
GI/hepatobiliary	2.96	1.70-5.14	<0.001	
Other	2.30	1.34-3.93	0.002	
CKD	0.75	0.49-1.15	0.2	
CAD	0.99	0.66-1.47	0.9	
Heart failure	1.50	0.92-2.43	0.1	
Diabetes	0.90	0.64-1.26	0.5	
Model 2: hs-cTnI, fully adjusted				0.595 (0.558-0.631)
hs-cTnI elevated (≥45.2 ng/L)	0.91	0.33-2.52	0.9	
Age (per y)	1.01	1.00-1.02	0.2	
Women	0.76	0.57-1.01	0.055	
Melanoma (reference)	1.00	Reference	—	
Lung	1.63	0.90-2.97	0.1	
Head and neck/cutaneous SCC	2.15	1.23-3.77	0.007	
Genitourinary	1.86	1.07-3.26	0.029	
GI/hepatobiliary	2.91	1.68-5.06	<0.001	
Other	2.29	1.34-3.93	0.002	
CKD	0.77	0.51-1.18	0.2	
CAD	1.00	0.67-1.48	>0.99	
Heart failure	1.66	1.03-2.67	0.037	
Diabetes	0.93	0.67-1.30	0.7	
Model 3: Concordance group, fully adjusted				0.609 (0.572-0.646)
TnT+/TnI−	1.31	0.97-1.76	0.077	
Both elevated	1.77	0.61-5.15	0.3	
TnI+/TnT−	—	—	—	
Age (per y)	1.01	0.99-1.02	0.3	
Women	0.80	0.60-1.07	0.1	
Melanoma (reference)	1.00	Reference	—	
Lung	1.58	0.86-2.89	0.1	
Head and neck/cutaneous SCC	2.21	1.26-3.87	0.006	
Genitourinary	1.84	1.05-3.22	0.033	
GI/hepatobiliary	2.94	1.69-5.12	<0.001	
Other	2.34	1.37-4.01	0.002	
CKD	0.77	0.50-1.17	0.2	
CAD	0.98	0.66-1.46	0.9	
Heart failure	1.49	0.92-2.41	0.1	
Diabetes	0.89	0.64-1.24	0.5	
Model 4: Concordance + continuous troponins				0.627 (0.591-0.663)
TnT+/TnI−	0.82	0.51-1.30	0.4	
Both elevated	0.98	0.15-6.49	>0.99	
TnI+/TnT−	—	—	—	
log(hs-cTnT), continuous	1.78	1.21-2.62	0.003	
log(hs-cTnI), continuous	0.78	0.62-0.99	0.041	
Age (per y)	1.00	0.99-1.02	0.6	
Women	0.82	0.61-1.10	0.2	
Melanoma (reference)	1.00	Reference	—	
Lung	1.69	0.91-3.11	0.094	
Head and neck/cutaneous SCC	2.34	1.33-4.11	0.003	
Genitourinary	1.96	1.11-3.44	0.020	
GI/hepatobiliary	3.25	1.85-5.69	<0.001	
Other	2.49	1.45-4.28	<0.001	
CKD	0.71	0.46-1.10	0.1	
CAD	1.06	0.71-1.57	0.8	
Heart failure	1.54	0.95-2.51	0.080	
Diabetes	0.85	0.61-1.19	0.3	

All models use N = 433 patients with baseline concordance data and survival follow-up and are adjusted for age, sex, cancer category, CKD, CAD, heart failure, and diabetes. Reference categories: hs-cTnT not elevated, hs-cTnI not elevated, male sex, melanoma (cancer category), and both normal (concordance group). Unadjusted and age- and sex-adjusted models are provided in Supplemental Table 5. C-statistics are Harrell’s C (global concordance) with 95% CI. There was no evidence of violation of the proportional hazards assumption (global Schoenfeld *P* = 0.45).

CAD = coronary artery disease; CKD = chronic kidney disease; GI = gastrointestinal; hs-cTnI = high-sensitivity cardiac troponin I; hs-cTnT = high-sensitivity cardiac troponin T; SCC = squamous cell carcinoma; TnI+/TnT− = elevated hs-cTnI with normal hs-cTnT; TnT+/TnI− = elevated hs-cTnT with normal hs-cTnI.

Benjamini-Hochberg correction was prespecified for secondary outcomes within each analytical family (correlates of discordance; Cox models); reported *P* values are unadjusted unless otherwise noted, and adjusted results are flagged where they alter inference.

### Missing data and sensitivity analyses

All key baseline covariates were complete after database verification of 34 values across 22 patients; no imputation was required. Six prespecified sensitivity analyses were performed: sex-specific URLs (both assays), LOD/2 and LOD/√2 substitution for below-LOD values, complete-case restriction to patients with all 3 timepoints, cancer-type and ICI-class subgroups, and extreme-outlier exclusion (troponin >10× URL).

### Software

Analyses were performed in R statistical software (version 4.5.3; R Core Team, 2026). A *P* value of less than 0.05 was considered statistically significant.

## Results

### Study group

Of 693 patients enrolled, 164 were excluded because of lack of residual blood, yielding 529 patients; of these, 433 (81.9%) had paired baseline measurements and formed the primary analytical cohort (Supplemental Figure 1).

Among the 433 patients with paired baseline measurements, the median age was 67 years (Q1-Q3: 59-73), 238 (55.0%) were men, and the most common cancers were genitourinary (18.9%), head and neck/cutaneous squamous cell carcinoma (17.3%), gastrointestinal/hepatobiliary (15.5%), melanoma (13.4%), and lung (13.4%) (Table 1). The predominant ICI class was anti-PD-1 (74.6%). Over a median follow-up of 30.1 months (95% CI: 29.4-30.8; reverse Kaplan-Meier), 231 patients died (Kaplan-Meier–estimated mortality, 49.3%; 95% CI: 44.3-53.9 at 24 months), 8 (3.5%) of whom had an adjudicated cardiac cause of death.

**Table 1:** Baseline Characteristics of the Analytical Cohort Overall and Stratified by Concordance Group

	Overall (N = 433)	Both Normal (n = 267)	TnT+/TnI− (n = 158)	Both Elevated (n = 6)	TnI+/TnT− (n = 2)	*P* Value
Age, y, median [Q1-Q3]	67.00 [59.00, 73.00]	65.00 [56.00, 70.00]	71.00 [65.00, 78.00]	70.00 [61.75, 74.50]	66.50 [63.25, 69.75]	<0.001
Women, n (%)	195 (45.0)	144 (53.9)	46 (29.1)	4 (66.7)	1 (50.0)	<0.001
Race, n (%)						0.30
AI/AN	5 (1.2)	4 (1.5)	1 (0.6)	0 (0.0)	0 (0.0)	
Asian	13 (3.0)	5 (1.9)	8 (5.1)	0 (0.0)	0 (0.0)	
Black	29 (6.8)	18 (6.8)	9 (5.8)	2 (33.3)	0 (0.0)	
White	369 (86.2)	230 (87.1)	133 (85.3)	4 (66.7)	2 (100.0)	
Other	12 (2.8)	7 (2.7)	5 (3.2)	0 (0.0)	0 (0.0)	
BMI, kg/m^2^, median [Q1-Q3]	27.12 [23.51, 31.95]	27.17 [23.63, 31.55]	26.91 [23.37, 32.33]	26.67 [20.41, 32.29]	29.55 [29.00, 30.10]	0.90
Hypertension, n (%)	237 (54.7)	121 (45.3)	110 (69.6)	5 (83.3)	1 (50.0)	<0.001
Diabetes, n (%)	88 (20.3)	40 (15.0)	47 (29.7)	1 (16.7)	0 (0.0)	0.002
CAD, n (%)	59 (13.6)	24 (9.0)	34 (21.5)	1 (16.7)	0 (0.0)	0.003
Heart failure, n (%)	33 (7.6)	7 (2.6)	24 (15.2)	2 (33.3)	0 (0.0)	<0.001
CKD, n (%)	54 (12.5)	16 (6.0)	37 (23.4)	0 (0.0)	1 (50.0)	<0.001
COPD, n (%)	40 (9.3)	24 (9.0)	15 (9.6)	1 (16.7)	0 (0.0)	0.60
Stroke, n (%)	16 (3.7)	9 (3.4)	7 (4.5)	0 (0.0)	0 (0.0)	0.70
PVD, n (%)	17 (3.9)	7 (2.6)	10 (6.4)	0 (0.0)	0 (0.0)	0.20
Hyperlipidemia, n (%)	205 (47.7)	106 (40.0)	95 (60.5)	4 (66.7)	0 (0.0)	<0.001
Smoking status, n (%)						0.094
Never	191 (44.1)	122 (45.7)	66 (41.8)	1 (16.7)	2 (100.0)	
Former	203 (46.9)	118 (44.2)	82 (51.9)	3 (50.0)	0 (0.0)	
Current	39 (9.0)	27 (10.1)	10 (6.3)	2 (33.3)	0 (0.0)	
Pack-years, median [Q1-Q3]	28.25 [15.00, 40.58]	30.00 [15.00, 45.00]	21.50 [10.00, 37.50]	24.40 [11.56, 33.92]	—	0.2
Cancer category, n (%)						—
Melanoma	58 (13.4)	40 (15.0)	18 (11.4)	0 (0.0)	0 (0.0)	
Lung	58 (13.4)	38 (14.2)	17 (10.8)	3 (50.0)	0 (0.0)	
Head and neck/cutaneous SCC	75 (17.3)	44 (16.5)	29 (18.4)	2 (33.3)	0 (0.0)	
Genitourinary	82 (18.9)	38 (14.2)	43 (27.2)	0 (0.0)	1 (50.0)	
GI/hepatobiliary	67 (15.5)	44 (16.5)	22 (13.9)	1 (16.7)	0 (0.0)	
Other	93 (21.5)	63 (23.6)	29 (18.4)	0 (0.0)	1 (50.0)	
ICI class, n (%)						0.1
Anti-PD-1	323 (74.6)	189 (70.8)	128 (81.0)	4 (66.7)	2 (100.0)	
Anti-PD-L1	67 (15.5)	51 (19.1)	14 (8.9)	2 (33.3)	0 (0.0)	
Anti-CTLA-4	4 (0.9)	3 (1.1)	1 (0.6)	0 (0.0)	0 (0.0)	
Combination	38 (8.8)	24 (9.0)	14 (8.9)	0 (0.0)	0 (0.0)	
Other	1 (0.2)	0 (0.0)	1 (0.6)	0 (0.0)	0 (0.0)	
hs-cTnT, baseline, ng/L, median [Q1-Q3]	11.00 [6.00, 19.00]	7.00 [6.00, 10.00]	22.00 [17.00, 29.00]	46.00 [26.25, 547.25]	10.50 [9.75, 11.25]	<0.001
hs-cTnI, baseline, ng/L, median [Q1-Q3]	2.52 [2.50, 5.59]	2.50 [2.50, 3.36]	4.61 [2.50, 9.64]	108.37 [79.55, 1892.87]	100.57 [85.57, 115.57]	<0.001

Values are median [Q1-Q3] or n (%). *P* values by Kruskal-Wallis (continuous) or Fisher exact test (categorical). Both elevated (n = 6) and TnI+/TnT− (n = 2) groups have very small sample sizes; interpret with caution. Race was missing for 5 patients; race percentages use the number with race recorded as the denominator.

AI/AN = American Indian/Alaska Native; BMI = body mass index; CAD = coronary artery disease; CKD = chronic kidney disease; COPD = chronic obstructive pulmonary disease; CTLA-4 = cytotoxic T-lymphocyte–associated protein 4; GI = gastrointestinal; hs-cTnI = high-sensitivity cardiac troponin I; hs-cTnT = high-sensitivity cardiac troponin T; ICI = immune checkpoint inhibitor; PD-1 = programmed cell death protein 1; PD-L1 = programmed death-ligand 1; PVD = peripheral vascular disease; SCC = squamous cell carcinoma.

### Troponin distributions and elevation rates

At baseline, median hs-cTnT was 11 ng/L (Q1-Q3: 6-19), with the Q1-Q3 interval spanning its URL of 14 ng/L, whereas median hs-cTnI was 2.52 ng/L (Q1-Q3: 2.50-5.59), well below its URL of 45.2 ng/L; 49.7% of hs-cTnI values fell below the LOD vs 22.4% for hs-cTnT values (Supplemental Figure 2). hs-cTnT was elevated (≥14 ng/L) in 37.9% vs 1.8% for hs-cTnI (≥45.2 ng/L), an approximately 20-fold difference (McNemar *P* < 0.001). The proportions were similar at 1 month (hs-cTnT, 40.1%; hs-cTnI, 3.6%).

### TnT/TnI concordance

Among 433 patients with paired baseline measurements, concordance classification was as follows: both normal, 267 (61.7%), TnT+/TnI− (discordant), 158 (36.5%), both elevated, 6 (1.4%), and TnI+/TnT− (reverse discordant), 2 (0.5%). Interassay agreement was near zero (κ = 0.036; 95% CI: −0.001 to 0.080) (Figure 1, Central Illustration).

**Figure 1:**
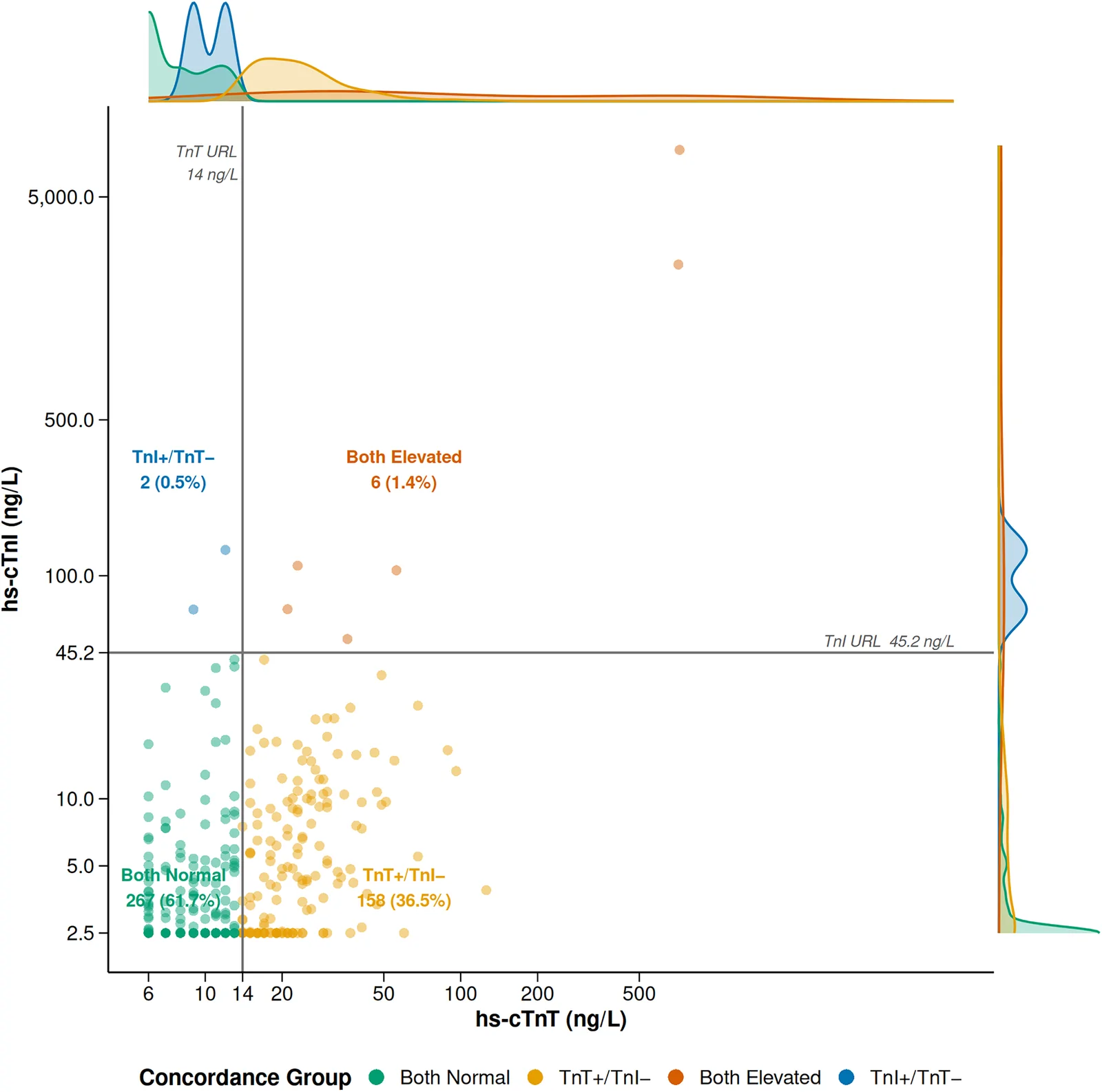
Hs-cTnT vs hs-cTnI at Baseline Scatter plot of paired baseline high-sensitivity cardiac Tropnin T (hs-cTnT) (x-axis) and high-sensitivity cardiac Tropnin I (hs-cTnI) (y-axis) measurements on log-log axes with marginal densities. Reference lines indicate the 99th-percentile URLs for hs-cTnT (14 ng/L) and hs-cTnI (45.2 ng/L). Values below the limit of detection were plotted at the limit of detection (hs-cTnT, 6 ng/L; hs-cTnI, 2.5 ng/L). Points and marginal densities are colored by concordance group, and quadrant labels show group sizes and percentages. TnI = troponin I; TnT = troponin T.

**Central Illustration:**
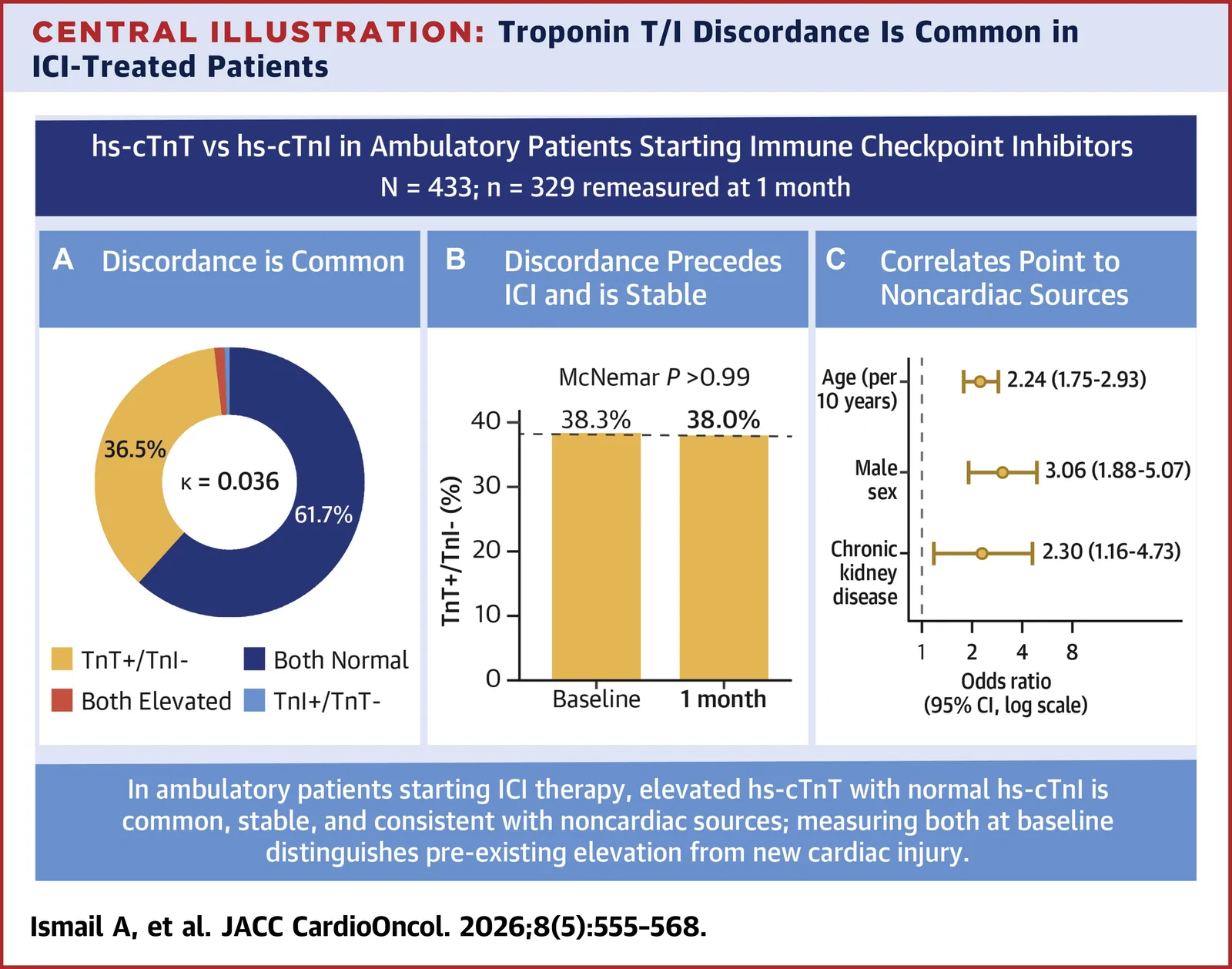
Troponin T/I Discordance Is Common in ICI-Treated Patients (A) Among 433 patients starting immune checkpoint inhibitor (ICI) therapy with paired baseline high-sensitivity cardiac troponin T (hs-cTnT) and high-sensitivity cardiac troponin I (hs-cTnI) measurements, 158 (36.5%) had elevated hs-cTnT with normal hs-cTnI (discordant), 267 (61.7%) had normal levels of both, and 8 (1.8%) had other patterns (κ = 0.036). (B) Among 329 patients measured at baseline and 1 month, discordance was unchanged (38.3% vs 38.0%; McNemar *P* > 0.99), supporting a stable baseline phenotype rather than an ICI-induced effect. (C) Independent correlates were older age (OR: 2.24 per 10 years), male sex (OR: 3.06), and chronic kidney disease (OR: 2.30), suggesting hs-cTnI provides greater cardiac specificity in these patients. OR = odds ratio; TnT+/TnI− = elevated hs-cTnT with normal hs-cTnI; TnI+/TnT− = elevated hs-cTnI with normal hs-cTnT.

Among the 329 patients classified at both timepoints, discordance prevalence was unchanged (38.3% vs 38.0%; McNemar *P* > 0.99); most patients with baseline TnT+/TnI− remained discordant at 1 month (Figure 2). Six-month classification, available only for a survivor-enriched subset, showed the same pattern (exploratory; Supplemental Figure 3, Supplemental Table 2). Discordant patients had median hs-cTnT of 22 ng/L (Q1-Q3: 17-29) and hs-cTnI of 4.6 ng/L (Q1-Q3: 2.5-9.6) (Table 1). Among discordant patients with available creatine phosphokinase (CPK; n = 22 of 158), 19 (86.4%) had normal levels (median, 70 U/L), similar to the both-normal group (*P* = 0.72).

**Figure 2:**
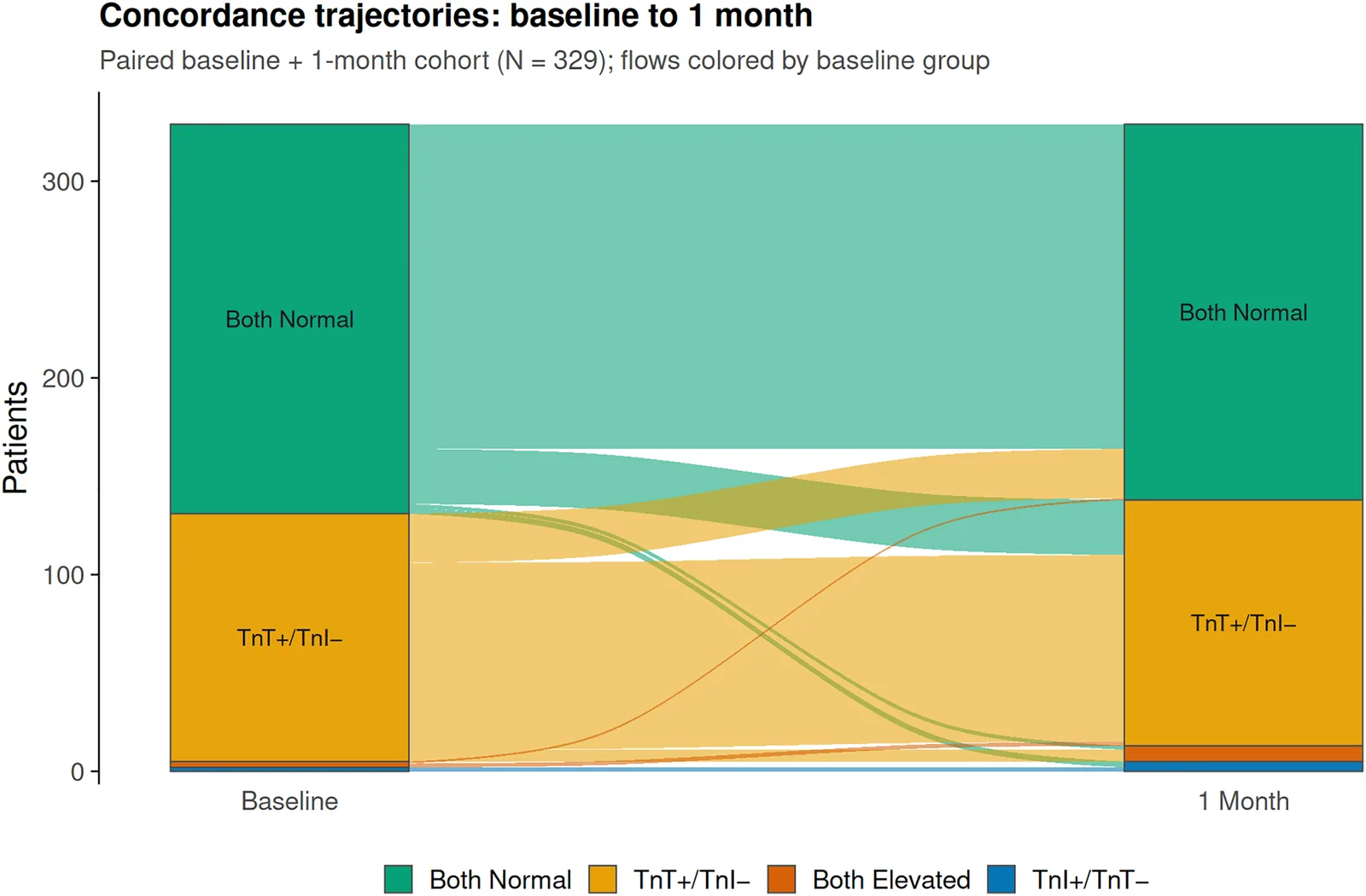
Stability of Troponin Concordance From Baseline to 1 Month Alluvial diagram of troponin concordance classification at baseline and 1 month among the 329 patients with paired high-sensitivity cardiac troponin T (hs-cTnT) and high-sensitivity cardiac troponin I (hs-cTnI) measurements at both timepoints. Vertical strata represent the 4 concordance groups; flows are colored by baseline group and represent individual patient trajectories. Elevated hs-cTnT with normal hs-cTnI (TnT+/TnI−) discordance prevalence was unchanged (38.3% vs 38.0%; McNemar *P* > 0.99); 67 of the 329 patients (20.4%) changed concordance group between timepoints, corresponding to the sum of the between-group flows shown. At both timepoints, the both-elevated and elevated hs-cTnI with normal hs-cTnT (TnI+/TnT−) groups each comprised <2% of patients.

### Longitudinal trajectories

Both biomarkers showed flat median trends from baseline to 1 month, with hs-cTnT remaining near its URL and hs-cTnI remaining well below its URL (Supplemental Figure 2).

In a linear mixed-effects model (1,588 observations, 433 patients), hs-cTnI concentrations were approximately 65% lower than hs-cTnT concentrations (β = −1.04 on the natural-log scale; 95% CI: −1.13 to −0.96; *P* < 0.001). Troponin concentrations did not change significantly from baseline to 1 month (*P* = 0.25), and the timepoint-by-biomarker interaction was not significant (*P* = 0.10), consistent with parallel, stable trajectories. Higher troponin concentrations were independently associated with older age (approximately 23% higher per decade; *P* < 0.001) and male sex (approximately 16% higher; *P* = 0.018).

### Clinical correlates of discordance

Given the stability of discordance, we examined which characteristics were associated with its presence at baseline, comparing TnT+/TnI− (n = 158) with both normal (n = 267) using multivariable logistic regression (Figure 3, Supplemental Table 3; all generalized variance inflation factors <1.08). Among 425 patients with complete data, 3 factors were independently associated with discordance: older age (OR: 2.24 per 10 years; 95% CI: 1.75-2.93; *P* < 0.001), male sex (OR: 3.06; 95% CI: 1.88-5.07; *P* < 0.001), and CKD (OR: 2.30; 95% CI: 1.16-4.73; *P* = 0.020); body mass index was not significant. Anti-PD-L1 therapy was associated with lower odds of discordance than anti-PD-1 (OR: 0.38; 95% CI: 0.17-0.82), which may reflect its disproportionate use in lung cancer, which had the lowest discordance rate (29.3%); no cancer type was independently significant. The C-statistic was 0.792 (95% CI: 0.747-0.837), and all 3 correlates remained significant after Benjamini-Hochberg correction.

**Figure 3:**
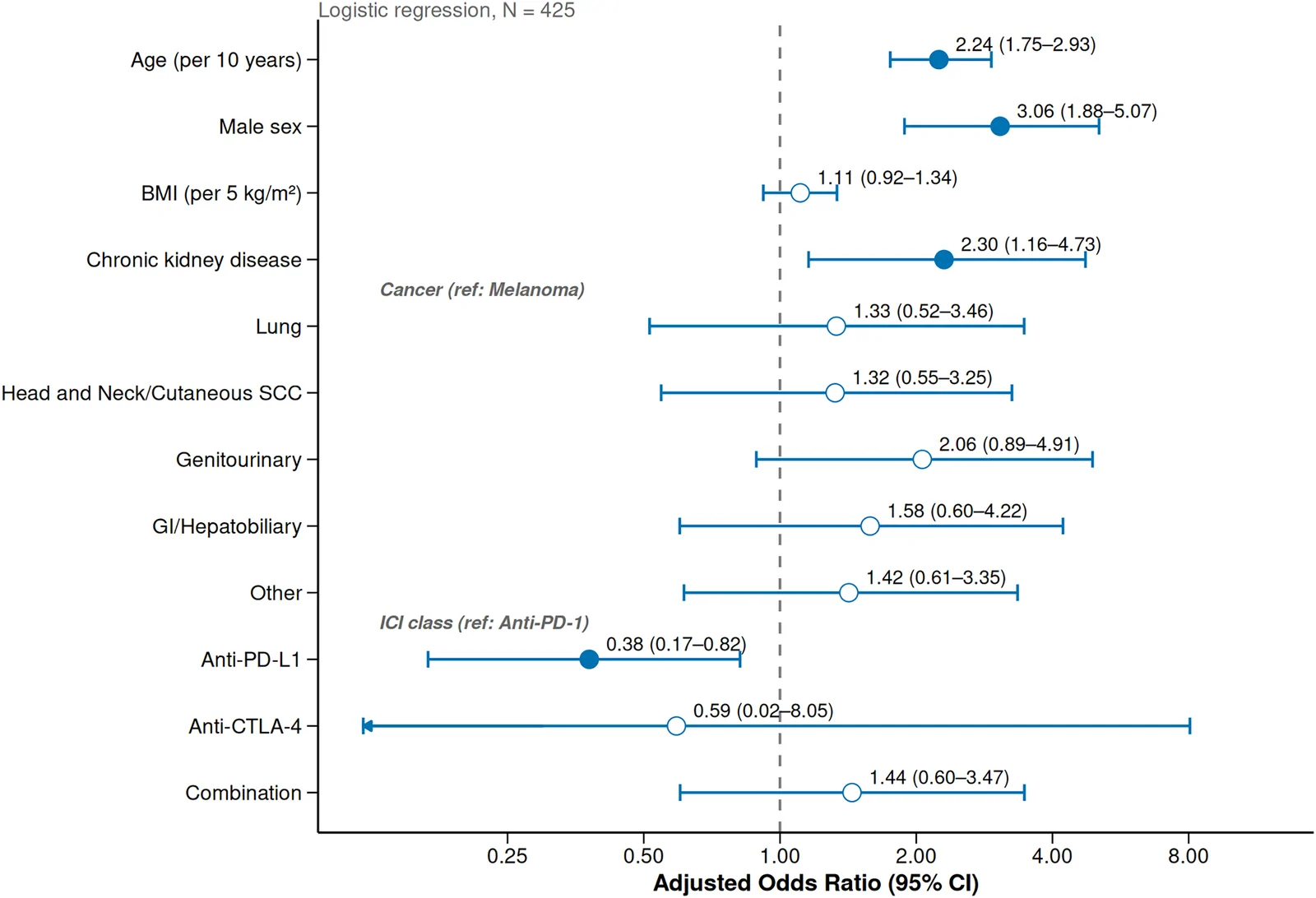
Clinical Correlates of Elevated hs-cTnT With Normal hs-cTnI Discordance Adjusted ORs with 95% CIs from multivariable logistic regression comparing elevated hs-cTnT with normal hs-cTnI discordance with both normal at baseline (N = 425). The model included age (per 10 years), sex, body mass index (per 5 kg/m^2^), chronic kidney disease, cancer category, and immune checkpoint inhibitor class. Reference categories are female sex, no chronic kidney disease, melanoma (cancer category), and anti-PD-1 (immune checkpoint inhibitor class). Filled circles denote *P* < 0.05 and open circles *P* ≥ 0.05. Full model results are in Supplemental Table 3. BMI = body mass index; CTLA = cytotoxic T-lymphocyte–associated protein; GI = gastrointestinal; hs-cTnI = high-sensitivity cardiac troponin I; hs-cTnT = high-sensitivity cardiac troponin T; ICI = immune checkpoint inhibitor; OR = odds ratio; PD = programmed cell death; SCC = squamous cell carcinoma.

In a sensitivity model adding coronary artery disease, heart failure, hypertension, diabetes, and smoking, associations with age, sex, and CKD were preserved. Heart failure emerged as an additional correlate (OR: 4.32; 95% CI: 1.61-12.78; *P* = 0.005). Smoking was not associated with discordance (Supplemental Table 3).

### Survival analysis

hs-cTnT elevation was associated with worse all-cause survival (median, 19.2 vs 29.2 months; log-rank *P* = 0.004) (Figure 4A). The both-elevated (n = 6) and reverse-discordant (n = 2) groups were too small to display as separate Kaplan-Meier strata; both-elevated estimates are reported in the Cox models (Table 2) for completeness but are imprecise, and TnI+/TnT− could not be estimated. Patients with TnT+/TnI− had worse survival than those with both normal (median, 19.2 vs 27.9 months; log-rank *P* = 0.007; Figure 4B).

**Figure 4:**
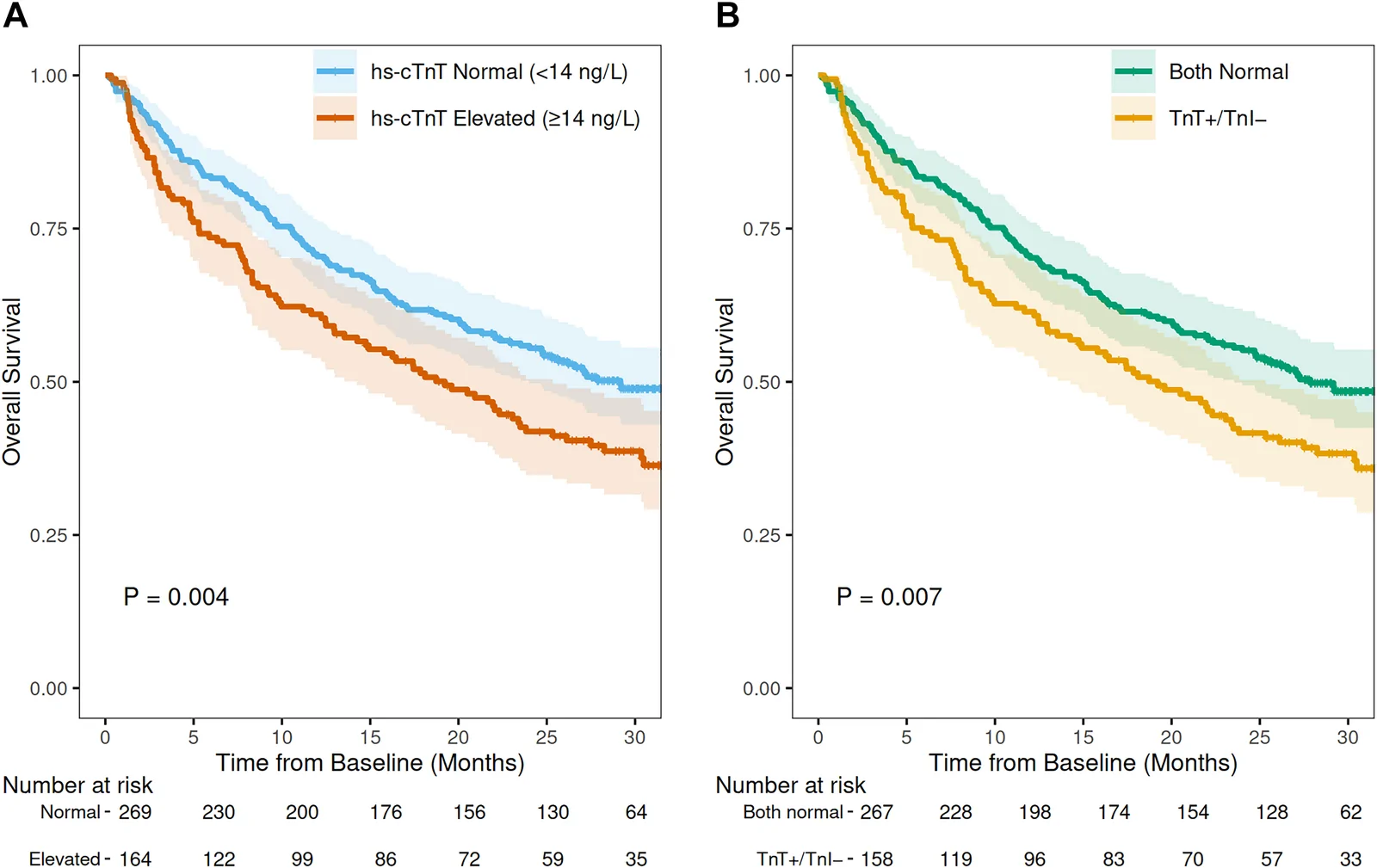
All-Cause Survival by Baseline Troponin Status in the Paired Cohort Kaplan-Meier curves in the paired-baseline cohort (N = 433), stratified by (A) high-sensitivity cardiac troponin T elevation (≥14 vs <14 ng/L), and (B) both normal vs elevated hs-cTnT with normal hs-cTnI (TnT+/TnI−) (discordant) at baseline (n = 425). The both-elevated (n = 6) and reverse-discordant (n = 2) groups were not included in (B) because of small group sizes. Curves are truncated at 30 months because of sparse risk sets beyond that point; risk tables are shown in A and B. *P* values are from the log-rank test. hs-cTnI = high-sensitivity cardiac troponin I; hs-cTnT = high-sensitivity cardiac troponin T.

In Cox models (Table 2), baseline hs-cTnT elevation was associated with mortality across all specifications (fully adjusted HR: 1.35; 95% CI: 1.00-1.81; *P* = 0.048). Baseline hs-cTnI elevation was not associated with mortality in any specification (unadjusted HR: 0.90; 95% CI: 0.33-2.45 [Supplemental Table 5]; fully adjusted HR: 0.91; 95% CI: 0.33-2.52 [Table 2]), although the small number of patients with elevated hs-cTnI (n = 8) limits precision. The association between TnT+/TnI− and mortality compared with both normal was not statistically significant (adjusted HR: 1.31; 95% CI: 0.97-1.76; *P* = 0.077) and was attenuated after adjustment for continuous troponin values (HR: 0.82). There was no evidence of violation of the proportional hazards assumption (global Schoenfeld *P* = 0.45) (Table 2).

Eight cardiac deaths occurred during follow-up, precluding meaningful inference from cause-specific or competing-risk analyses.

### Sensitivity analyses

TnT+/TnI− discordance was preserved across all prespecified sensitivity analyses, with baseline rates ranging from 25.4% to 36.7% compared with the 36.5% primary analysis rate (Supplemental Table 4). Applying sex-specific URLs to hs-cTnI alone produced negligible change (36.7%); applying them to both assays reduced but did not eliminate discordance (25.4% at baseline and 1 month), driven by reclassification of men with hs-cTnT of 14-21 ng/L, with older age and CKD remaining the dominant correlates (both *P* < 0.01). Below-LOD substitution (LOD/2 or LOD/√2) did not alter binary concordance, and exclusion of 2 extreme outliers (>10× URL) left the concordance distribution unchanged and yielded a similar Cox effect estimate (HR: 1.30; *P* = 0.087). Discordance varied by subgroup and was highest in genitourinary (52.4%) and lowest in lung cancer (29.3%); discordance was also lower with anti-PD-L1 (20.9%) than with anti-PD-1 (39.6%) therapy. In an exploratory complete-case cohort of 119 patients with all 3 timepoints, discordance was stable (32.8%, 32.8%, and 36.1% at baseline, 1 month, and 6 months, respectively); lower mortality in this subset (24-month Kaplan-Meier–estimated mortality, 36.7% vs 52.0%) is consistent with survivorship bias, and 6-month results are reported as exploratory (Supplemental Figure 3, Supplemental Table 2).

## Discussion

In this prospective cohort with paired hs-cTnI and hs-cTnT measurements at the start of ICI therapy, 4 findings emerged. First, over one-third of ambulatory patients had elevated hs-cTnT with normal hs-cTnI. Second, this discordance was present before ICI exposure and stable through 1 month, consistent with a chronic, noncardiac source. Third, the discordance was associated with older age, male sex, and CKD, factors linked to sarcopenia, chronic organ dysfunction, and reduced troponin clearance. Fourth, hs-cTnT was associated with all-cause mortality, and adjustment for continuous troponin attenuated the association between discordance and mortality, pointing to comorbidity burden rather than occult cardiac injury.

### Biological basis of discordance

Frequent hs-cTnT elevation in ambulatory ICI-treated patients without acute cardiac disease may arise from several noncardiac sources. *TNNT2*, normally silenced in adult skeletal muscle, is re-expressed in diseased muscle without re-expression of *TNNI3*.^20^^,^^21^^,^^31^ Isolated hs-cTnT elevation is well documented in skeletal myopathies and inflammatory myositis.^21^ The lower rate in our cohort (approximately 37% vs approximately 69% in overt myopathy) is consistent with subclinical muscle involvement, which is plausible in a study group enriched for sarcopenia and cachexia.^32^ Reduced renal clearance in CKD^22^ and chronic myocardial turnover^19^ may also contribute. Reverse discordance was rare (0.5%), consistent with the greater cardiac specificity of TnI. We did not directly measure these sources, so mechanistic attribution remains inferential.

Whether ICI therapy itself induces hs-cTnT elevation without clinical myotoxicity, a concern raised by TnT persistence for months after clinical ICI myotoxicity,^33^ is not supported by our data: discordance preceded ICI exposure, CPK levels were normal in 19 of 22 discordant patients with available measurements, and prevalence was stable. If ICI therapy were driving subclinical injury, rising discordance or diverging trajectories would be expected; neither occurred. By contrast, in manifest ICI myocarditis,^5^^,^^27^, ^28^, ^29^ TnT-to-TnI ratios reached approximately 15, far above our discordant median ratio of 5.2 (Q1-Q3: 2.6-6.9), distinguishing the chronic ambulatory phenotype from acute injury.

### Prognostic interpretation

The finding that hs-cTnT is associated with all-cause mortality (HR: 1.35), whereas hs-cTnI is not (HR: 0.91), may have 2 complementary explanations. First, hs-cTnI was elevated in only 8 patients, too few to detect an association if one existed. Second, the association with hs-cTnT is unlikely to reflect primarily acute cardiac injury: only 8 of 231 deaths (3.5%) were cardiac. Because hs-cTnT may reflect cardiac injury, skeletal muscle turnover, reduced clearance, and comorbidity, it may function as a composite marker of frailty and disease burden. Consistent with this interpretation, the association between discordance and mortality was attenuated after adjustment for continuous troponin values, and the correlates of discordance (age, sex, and CKD) are themselves established mortality risk factors. These associations align with Shibutani et al^33^ and with the “cardiomuscular” biomarker concept.^30^^,^^34^^,^^35^ A troponin associated with all-cause mortality when cardiac events are too rare to evaluate may lead to over-triage if interpreted without consideration of cardiac specificity.

### Clinical implications for troponin surveillance

Our baseline hs-cTnT elevation rate (37.9%) was similar to the approximately 40% reported by Tomsitz et al,^36^ whereas cTnI-based surveillance detects far fewer elevations. In another cohort that did not exclude myocarditis, only 3% had elevated hs-cTnI at baseline, similar to the 1.8% reported here.^37^

The discordance pattern suggests that many hs-cTnT elevations may not reflect acute cardiac injury. Because an ICI myocarditis diagnosis triggers corticosteroids, immunotherapy discontinuation, and sometimes secondary immunosuppression, a nonspecific biomarker may lead to unnecessary intervention and dilute attention when myocarditis occurs.^30^^,^^38^^,^^39^ Because we excluded adjudicated myocarditis, our findings speak to specificity rather than comparative sensitivity: hs-cTnI is the more cardiac-specific troponin, and paired baseline measurement may help contextualize later elevations. A new rise in hs-cTnI or a rise above a stable hs-cTnT baseline may be more interpretable than a single mid-treatment value. This complements the Vasbinder et al noncardiac biomarker strategy, in which CPK, aspartate aminotransferase, and alanine aminotransferase rise a median of 20 days before ICI myocarditis (CPK sensitivity, 99%).^7^

These findings do not dismiss hs-cTnT. Where it is used exclusively, recognizing the high baseline prevalence and stability of isolated elevation may reduce overinterpretation, and a known baseline may make an acute rise more conspicuous. Because myocarditis and myositis frequently coexist in ICI toxicity,^28^^,^^29^ hs-cTnT’s sensitivity to skeletal muscle involvement may help flag combined cardiomuscular injury. Conversely, anti-cTnI antibodies, detectable in up to half of patients with ICI myocarditis in one series and capable of prolonging clearance and producing cross-assay interference,^39^ warrant vigilance for false-negative hs-cTnI results when suspicion is high.

Together with the converging mechanistic,^20^^,^^21^^,^^31^ cardiomuscular biomarker,^30^^,^^35^ and prospective surveillance^36^^,^^37^ literature, these data may inform how guidelines and future studies address the currently unspecified choice of troponin assay for ICI cardiac surveillance.

### Study limitations

This single-center study used 1 hs-cTnI and 1 hs-cTnT assay (Siemens Atellica and Roche Elecsys, respectively); findings may not generalize to other platforms, and interassay differences not attributable to myositis have been described in cardio-oncology cohorts.^40^^,^^41^ Because biomarkers were measured in residual samples and did not influence clinical care, the cohort captures an unselected ambulatory study group free of test-ordering bias; the trade-off is that biomarker behavior during interim hospitalizations was not captured. Follow-up beyond 1 month was limited and survivor-enriched, so longitudinal inferences rest on the baseline-to-1-month interval. The small numbers of hs-cTnI elevations and cardiac deaths preclude prognostic conclusions about hs-cTnI, and the concordance-based mortality association is hypothesis-generating. Multicenter confirmation with adjudicated cardiac events is needed.

## Conclusions

hs-cTnI and hs-cTnT are not interchangeable in ambulatory ICI-treated patients: isolated hs-cTnT elevation in over one-third of this cohort preceded ICI exposure and is consistent with chronic noncardiac sources. hs-cTnI is the more cardiac-specific assay, and paired baseline measurements contextualize later elevations when myocarditis is suspected.

Perspectives **COMPETENCY IN MEDICAL KNOWLEDGE:** In ambulatory patients receiving ICI therapy, over one-third had elevated hs-cTnT with normal hs-cTnI, a pattern present before ICI exposure, stable through 1 month, associated with older age, male sex, and chronic kidney disease, and consistent with chronic noncardiac sources rather than acute cardiac injury.

**TRANSLATIONAL OUTLOOK:** Because the diagnosis of ICI-associated myocarditis drives high-stakes treatment decisions, the cardiac specificity of the surveillance biomarker may affect diagnostic accuracy. hs-cTnI is the more cardiac-specific troponin, and paired baseline hs-cTnT and hs-cTnI measurements may help contextualize later elevations. These data may inform how guidelines and future studies address the currently unspecified choice of troponin assay for ICI cardiac surveillance.

## Funding Support and Author Disclosures

This study was supported by the Rogel Cancer Center Discovery Award at the University of Michigan and a donor gift from John Janitz. High-sensitivity cardiac troponin I (hs-cTnI) assays were measured by Siemens Healthineers, which did not fund the study and had no role in its design, data analysis, interpretation, or the decision to submit for publication. Dr Hayek is the guarantor of this work and, as such, has full access to all the data in the study and takes responsibility for the integrity of the data and the accuracy of the data analysis. The authors have reported that they have no relationships relevant to the contents of this paper to disclose.
