# Supplementary material for: High-Sensitivity Cardiac Troponin T and I Discordance in Patients Receiving Immune Checkpoint Inhibitors
Source: JACC CardioOncol. 2026 Oct 20;8(5):555–68. doi: 10.1016/j.jaccao.2026.07.012 (PMC13699023; doi:10.1016/j.jaccao.2026.07.012)
Supplement: Supplementary file 1 [file mmc1.docx]

Supplementary Materials

High-Sensitivity Cardiac Troponin T and I Discordance in Patients Receiving Immune Checkpoint Inhibitors

**Contents**

- Figure S1. Patient flow diagram
- Figure S2. Distributions of hs-cTnT and hs-cTnI across timepoints
- Figure S3. Exploratory six-month concordance trajectories
- Table S1. Baseline characteristics of excluded patients versus the analytic cohort
- Table S2. Baseline characteristics by availability of 6-month troponin data
- Table S3. Multivariable logistic regression: clinical correlates of discordance
- Table S4. TnT+/TnI− discordance by subgroup
- Table S5. Cox proportional hazards models for all-cause mortality (unadjusted and age/sex-adjusted)

# Supplementary Figures

**Figure S1. Patient Flow Diagram.** Derivation of the analytic cohort. Of 693 patients enrolled, 164 were excluded for lack of residual blood at any timepoint, yielding a 529-patient analytic cohort; 433 (81.9%) had paired baseline hs-cTnT and hs-cTnI measurements and constituted the primary analytic cohort. Abbreviations: hs-cTnI, high-sensitivity cardiac troponin I; hs-cTnT, high-sensitivity cardiac troponin T; ICI, immune checkpoint inhibitor.

**Figure S2. Distributions of hs-cTnT and hs-cTnI Across Timepoints.** Violin-and-box plots of hs-cTnT and hs-cTnI on a log10 scale at baseline, 1 month, and 6 months. Dashed lines mark each assay’s upper reference limit (URL; hs-cTnT 14 ng/L, hs-cTnI 45.2 ng/L) and limit of detection (LOD; hs-cTnT 6 ng/L, hs-cTnI 2.5 ng/L). hs-cTnT tracks near its URL while hs-cTnI remains well below its URL at every timepoint. Abbreviations: LOD, limit of detection; URL, upper reference limit.

**Figure S3. Exploratory Six-Month Concordance Trajectories.** Alluvial diagram of troponin concordance classification at baseline, 1 month, and 6 months among the 119 patients with paired hs-cTnT and hs-cTnI measurements at all three timepoints. Because only patients well enough to provide 6-month samples are represented, this subset is survivor-enriched and the 6-month timepoint is reported as exploratory only. Flows are colored by baseline concordance group. Abbreviations: hs-cTnI, high-sensitivity cardiac troponin I; hs-cTnT, high-sensitivity cardiac troponin T.

# Supplementary Tables

**Table S1. Baseline Characteristics of Excluded Patients (N = 164) Versus the Analytic Cohort (N = 433)**

| **Variable** | **Analytic Cohort (N = 433)** | **Excluded (N = 164)** | **p Value** |
| --- | --- | --- | --- |
| Age, years, median [Q1–Q3] | 67 [59-73] | 65 [56-72] | 0.096 |
| Male sex, n (%) | 238 (55.0%) | 105 (63.6%) | 0.057 |
| BMI, kg/m², median [Q1–Q3] | 27.1 [23.5-32.0] | 26.4 [22.9-31.8] | 0.4 |
| Hypertension, n (%) | 237 (54.7%) | 92 (55.8%) | 0.8 |
| Diabetes, n (%) | 88 (20.3%) | 32 (19.4%) | 0.9 |
| CAD, n (%) | 59 (13.6%) | 33 (20.0%) | 0.066 |
| Heart failure, n (%) | 33 (7.6%) | 15 (9.1%) | 0.7 |
| CKD, n (%) | 54 (12.5%) | 29 (17.6%) | 0.1 |
| COPD, n (%) | 40 (9.2%) | 19 (11.5%) | 0.5 |

p values by Wilcoxon rank-sum test (continuous) or chi-squared/Fisher’s exact test (categorical). The analytic cohort comprises the 433 patients with paired baseline hs-cTnT and hs-cTnI; excluded patients (N = 164) had no residual blood at any timepoint. An additional 96 patients had troponin measured at ≥ 1 timepoint but lacked paired baseline values and are not shown.

Abbreviations: BMI, body mass index; CAD, coronary artery disease; CKD, chronic kidney disease; COPD, chronic obstructive pulmonary disease.

**Table S2. Baseline Characteristics by Availability of 6-Month Troponin Data**

| **Variable** | **With 6-Month Data (N = 171)** | **Without 6-Month Data (N = 358)** | **p Value** |
| --- | --- | --- | --- |
| Age, years, median [Q1–Q3] | 66 [57-71] | 67 [60-74] | 0.020 |
| Male sex, n (%) | 87 (50.9%) | 216 (60.3%) | 0.050 |
| BMI, kg/m², median [Q1–Q3] | 28.4 [24.1-32.3] | 26.8 [23.1-32.0] | 0.081 |
| Hypertension, n (%) | 88 (51.5%) | 203 (56.7%) | 0.3 |
| Diabetes, n (%) | 34 (19.9%) | 77 (21.5%) | 0.8 |
| CAD, n (%) | 15 (8.8%) | 57 (15.9%) | 0.035 |
| Heart failure, n (%) | 9 (5.3%) | 29 (8.1%) | 0.3 |
| CKD, n (%) | 25 (14.6%) | 40 (11.2%) | 0.3 |
| COPD, n (%) | 12 (7.0%) | 37 (10.3%) | 0.3 |
| Baseline hs-cTnT, ng/L, median [Q1–Q3] | 10.0 [6.0-17.0] | 11.0 [7.0-20.0] | 0.069 |
| Baseline hs-cTnI, ng/L, median [Q1–Q3] | 2.5 [2.5-4.8] | 3.1 [2.5-6.8] | 0.005 |
| Baseline TnT+/TnI− discordance, n (%) | 57 (35.6%) | 101 (37.0%) | 0.7 |

p values by Wilcoxon rank-sum test (continuous) or chi-squared/Fisher’s exact test (categorical).

Abbreviations: BMI, body mass index; CAD, coronary artery disease; CKD, chronic kidney disease; COPD, chronic obstructive pulmonary disease; hs-cTnI, high-sensitivity cardiac troponin I; hs-cTnT, high-sensitivity cardiac troponin T.

**Table S3. Multivariable Logistic Regression: Clinical Correlates of TnT+/TnI− Discordance at Baseline (Full Model Coefficients)**

**Primary Model (C-statistic = 0.792; 95% CI 0.747–0.837)**

| **Variable** | **OR** | **95% CI** | **p Value** |
| --- | --- | --- | --- |
| Age (per 10 years) | 2.24 | 1.75–2.93 | <0.001 |
| Male sex | 3.06 | 1.88–5.07 | <0.001 |
| BMI (per 5 kg/m²) | 1.11 | 0.92–1.34 | 0.3 |
| CKD | 2.30 | 1.16–4.73 | 0.020 |
| Cancer category (ref: Melanoma) |  |  |  |
| Lung | 1.33 | 0.52–3.46 | 0.6 |
| Head & Neck/Cutaneous SCC | 1.32 | 0.55–3.25 | 0.5 |
| Genitourinary | 2.06 | 0.89–4.91 | 0.096 |
| GI/Hepatobiliary | 1.58 | 0.60–4.22 | 0.4 |
| Other | 1.42 | 0.61–3.35 | 0.4 |
| ICI class (ref: Anti-PD-1) |  |  |  |
| Anti-PD-L1 | 0.38 | 0.17–0.82 | 0.016 |
| Anti-CTLA-4 | 0.59 | 0.02–8.05 | 0.7 |
| Combination | 1.44 | 0.60–3.47 | 0.4 |

**Full Model (C-statistic = 0.811; 95% CI 0.769–0.854)**

| **Variable** | **OR** | **95% CI** | **p Value** |
| --- | --- | --- | --- |
| Age (per 10 years) | 2.08 | 1.59–2.77 | <0.001 |
| Male sex | 3.12 | 1.87–5.30 | <0.001 |
| BMI (per 5 kg/m²) | 1.05 | 0.86–1.27 | 0.6 |
| CKD | 1.63 | 0.78–3.49 | 0.2 |
| CAD | 1.15 | 0.56–2.36 | 0.7 |
| Heart failure | 4.32 | 1.61–12.78 | 0.005 |
| Hypertension | 1.39 | 0.83–2.35 | 0.2 |
| Diabetes | 1.66 | 0.92–2.99 | 0.092 |
| Smoking (ref: Never) |  |  |  |
| Former | 0.84 | 0.49–1.41 | 0.5 |
| Current | 0.69 | 0.26–1.69 | 0.4 |
| Cancer category (ref: Melanoma) |  |  |  |
| Lung | 1.27 | 0.45–3.64 | 0.7 |
| Head & Neck/Cutaneous SCC | 1.14 | 0.44–2.94 | 0.8 |
| Genitourinary | 2.02 | 0.84–4.98 | 0.1 |
| GI/Hepatobiliary | 1.54 | 0.56–4.29 | 0.4 |
| Other | 1.41 | 0.59–3.45 | 0.4 |
| ICI class (ref: Anti-PD-1) |  |  |  |
| Anti-PD-L1 | 0.32 | 0.14–0.72 | 0.008 |
| Anti-CTLA-4 | 0.80 | 0.03–9.92 | 0.9 |
| Combination | 1.34 | 0.54–3.32 | 0.5 |

Both models compare TnT+/TnI− (n = 158) versus Both Normal (n = 267) using multivariable logistic regression. The primary model includes age, sex, BMI, CKD, cancer category, and ICI class. The full model adds CAD, heart failure, hypertension, diabetes, and smoking status.

Abbreviations: BMI, body mass index; CAD, coronary artery disease; CI, confidence interval; CKD, chronic kidney disease; ICI, immune checkpoint inhibitor; OR, odds ratio; PD-1, programmed cell death protein 1; PD-L1, programmed death-ligand 1.

**Table S4. TnT+/TnI− Discordance by Subgroup**

**Panel A. Cancer Type**

| **Subgroup** | **N** | **Discordant, n** | **Discordant, %** |
| --- | --- | --- | --- |
| Genitourinary | 82 | 43 | 52.4 |
| Head & Neck/Cutaneous SCC | 75 | 29 | 38.7 |
| GI/Hepatobiliary | 67 | 22 | 32.8 |
| Other | 93 | 29 | 31.2 |
| Melanoma | 58 | 18 | 31.0 |
| Lung | 58 | 17 | 29.3 |

**Panel B. ICI Class**

| **Subgroup** | **N** | **Discordant, n** | **Discordant, %** |
| --- | --- | --- | --- |
| Anti-PD-1 | 323 | 128 | 39.6 |
| Combination | 38 | 14 | 36.8 |
| Anti-CTLA-4 | 4 | 1 | 25.0 |
| Anti-PD-L1 | 67 | 14 | 20.9 |
| Other | 1 | 1 | 100.0 |

Discordance defined as hs-cTnT ≥14 ng/L with hs-cTnI <45.2 ng/L at baseline among patients with paired measurements (N = 433). Sorted by descending discordance rate within each panel. The Panel B "Other" category (N = 1) should be interpreted with caution.

Abbreviations: hs-cTnI, high-sensitivity cardiac troponin I; hs-cTnT, high-sensitivity cardiac troponin T; ICI, immune checkpoint inhibitor; PD-1, programmed cell death protein 1; PD-L1, programmed death-ligand 1.

**Table S5. Cox Proportional Hazards Models for All-Cause Mortality: Unadjusted and Age- and Sex-Adjusted Specifications**

| **Model / Variable** | **HR** | **95% CI** | **P Value** | **C-Statistic** |
| --- | --- | --- | --- | --- |
| **Model 1a: hs-cTnT, unadjusted** |  |  |  | 0.546 (0.513–0.578) |
| hs-cTnT elevated (≥14 ng/L) | 1.46 | 1.13–1.89 | 0.004 |  |
| **Model 1b: hs-cTnI, unadjusted** |  |  |  | 0.500 (0.488–0.511) |
| hs-cTnI elevated (≥45.2 ng/L) | 0.90 | 0.33–2.45 | 0.8 |  |
| **Model 2a: hs-cTnT + age + sex** |  |  |  | 0.559 (0.520–0.598) |
| hs-cTnT elevated (≥14 ng/L) | 1.33 | 1.00–1.76 | 0.047 |  |
| Age (per year) | 1.01 | 0.99–1.02 | 0.3 |  |
| Female sex | 0.82 | 0.62–1.07 | 0.1 |  |
| **Model 2b: hs-cTnI + age + sex** |  |  |  | 0.543 (0.505–0.581) |
| hs-cTnI elevated (≥45.2 ng/L) | 0.93 | 0.34–2.54 | 0.9 |  |
| Age (per year) | 1.01 | 1.00–1.02 | 0.087 |  |
| Female sex | 0.77 | 0.59–1.00 | 0.049 |  |

Cox proportional hazards models for all-cause mortality (N=433). Unadjusted (Models 1a, 1b) and age- and sex-adjusted (Models 2a, 2b) specifications; the corresponding fully adjusted models are shown in Table 2 of the main text. Reference: troponin not elevated. C-statistics are Harrell’s C with 95% CI. Abbreviations: CI, confidence interval; HR, hazard ratio; hs-cTnI, high-sensitivity cardiac troponin I; hs-cTnT, high-sensitivity cardiac troponin T.
